# m^6^A Regulates Liver Metabolic Disorders and Hepatogenous Diabetes

**DOI:** 10.1016/j.gpb.2020.06.003

**Published:** 2020-11-05

**Authors:** Yuhuan Li, Qingyang Zhang, Guanshen Cui, Fang Zhao, Xin Tian, Bao-Fa Sun, Ying Yang, Wei Li

**Affiliations:** 1 State Key Laboratory of Stem Cell and Reproductive Biology, Institute of Zoology, Chinese Academy of Sciences, Beijing 100101, China; 2 University of Chinese Academy of Sciences, Beijing 100101, China; 3 CAS Key Laboratory of Genomic and Precision Medicine, Collaborative Innovation Center of Genetics and Development, College of Future Technology, Beijing Institute of Genomics, Chinese Academy of Sciences, Beijing 100101, China; 4 China National Center for Bioinformation, Beijing 100101, China; 5 Sino-Danish College, University of Chinese Academy of Sciences, Beijing 101408, China; 6 Department of Pharmacy, The First Affiliated Hospital of Zhengzhou University, Zhengzhou 450052, China; 7 Institute for Stem Cell and Regeneration, Chinese Academy of Sciences, Beijing 100101, China

**Keywords:** Mettl3, RNA methylation, High fat diet, Insulin resistance, *Lpin1*

## Abstract

*N*^6^-methyladenosine (m^6^A) is one of the most abundant modifications on mRNAs and plays important roles in various biological processes. The formation of m^6^A is catalyzed by a methyltransferase complex (MTC) containing a key factor methyltransferase-like 3 (**Mettl3**). However, the functions of Mettl3 and m^6^A modification in hepatic lipid and glucose metabolism remain unclear. Here, we showed that both Mettl3 expression and m^6^A level increased in the livers of mice with **high fat diet** (HFD)-induced metabolic disorders. Overexpression of *Mettl3* aggravated HFD-induced liver metabolic disorders and **insulin resistance**. In contrast, hepatocyte-specific knockout of *Mettl3* significantly alleviated HFD-induced metabolic disorders by slowing weight gain, reducing lipid accumulation, and improving insulin sensitivity. Mechanistically, Mettl3 depletion-mediated m^6^A loss caused extended RNA half-lives of metabolism-related genes, which consequently protected mice against HFD-induced metabolic syndrome. Our findings reveal a critical role of Mettl3-mediated m^6^A in HFD-induced metabolic disorders and hepatogenous diabetes.

## Introduction

As the most prevalent mRNA modification in eukaryotes [1], *N*^6^-methyladenosine (m^6^A) is catalyzed by a methyltransferase complex (MTC). MTC is composed of methyltransferase-like 3 (Mettl3), methyltransferase-like 14 (Mettl14), and wilms’ tumor 1-associating protein (Wtap), among which Mettl3 functions as the catalytic subunit [2], [3]. m^6^A methylation can be reversed by at least two ‘eraser’ enzymes, fat-mass and obesity-associated protein (Fto) and α-ketoglutarate-dependent dioxygenase alkB homolog 5 (Alkbh5) [4], [5]. And m^6^A is mainly recognized by YTH domain-containing family ‘reader’ proteins (Ythdfs) [6], [7], [8], [9], [10]. As the most abundant and reversible modification on mRNAs, m^6^A has been proved to play key roles in all fundamental aspects of mRNA metabolism, such as RNA stability [6], RNA splicing [8], and mRNA translation efficiency [7], [9], [10], [11]. Many essential biological processes are known to be regulated by m^6^A, including cell fate determination [12], [13], embryonic development [13], [14], [15], and tumorigenesis [16].

As the major site of fatty acid disposal, the main source of endogenous glucose production, and the primary site of insulin degradation, liver plays a central role in the regulation of lipid and glucose metabolism [17]. Unhealthy diet habits can result in liver metabolic disorders, followed by whole-body insulin resistance [17]. Several studies have revealed that m^6^A modulation of mRNA expression is involved in obesity [18] and liver metabolism [19], [20], and plays an important role in the maintenance and progression of liver diseases [21], [22], [23]. For instance, a significant increase in *FTO* mRNA and protein levels has been found in the liver of non-alcoholic fatty liver disease (NAFLD) patients [24]. Elevated levels of *Fto* mRNA and protein can also be found in a NAFLD rat, which was involved in oxidative stress and lipid deposition [25]. Knockdown of *Mettl3* or *Ythdf2 in vitro* increased the expression and stability of peroxisome proliferator activator receptor α (*Pparα*) mRNA, and then led to reduced accumulation of lipids [20]. A recent study showed that Mettl3 inhibited hepatic insulin sensitivity via m^6^A located in fatty acid synthase *(Fasn)* mRNA and promoted fatty acid metabolism [26]. All these studies indicated the important roles of m^6^A in liver metabolic diseases. However, the underlying mechanisms and pathways by which Mettl3-mediated m^6^A methylation affects liver metabolism are still not fully elucidated.

In the present work, we demonstrated that the m^6^A methyltransferase Mettl3 and m^6^A level were consistently up-regulated in the liver of mice after feeding high fat diet (HFD). Adeno-associated virus (AAV)-mediated liver-specific overexpression of *Mettl3* aggravated HFD-induced liver metabolic disorders and insulin resistance. In turn, we specifically inactivated Mettl3 in the mouse liver using *Alb*-Cre-mediated *Mettl3* conditional knockout (Mettl3^cKO^) model and confirmed that Mettl3 depletion protected mice against HFD-induced liver metabolic disorders and insulin resistance. Furthermore, mechanism analysis suggested that *Mettl3* deletion altered the expression pattern of hepatic lipid and glucose metabolic genes, and particularly extended the mRNA half-life of an important regulator of liver metabolism, *Lpin1*. Together, these findings reveal the critical role of Mettl3-mediated m^6^A modification in HFD-induced liver metabolic disorders and hepatogenous diabetes, supporting that m^6^A could be used as a potential therapeutic and diagnostic target for hepatic diseases.

## Results

### ***Mettl3*** expression and m^6^A level increased in HFD mice

**Figure 1:**
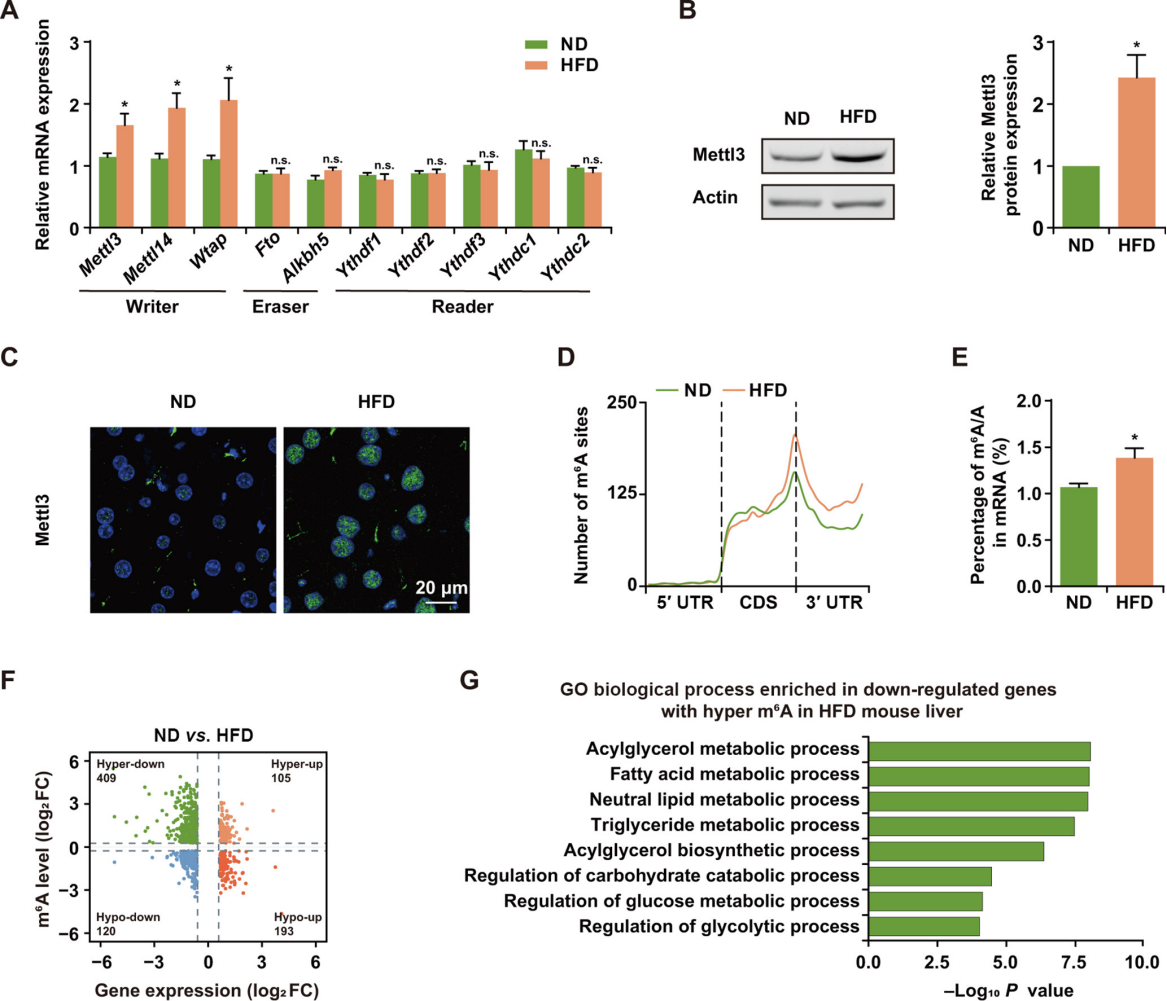
***Mettl3* expression and m^6^A level increased in HFD mice** **A.** qRT-PCR analysis of the expression of *Mettl3*, *Mettl14*, *Wtap*, *Fto*, *Alkbh5*, *Ythdf1*, *Ythdf2*, *Ythdf3*, *Ythdc1*, and *Ythdc2* in the livers of ND and HFD mice. *Ubc* served as the internal control. *n* = 5. **B.** Western blotting detection and quantification of Mettl3 protein expression in the livers of ND and HFD mice. Actin was used as the loading control. *n* = 3. **C.** Immunostaining of Mettl3 (green) in the livers of ND and HFD mice. Scale bar, 20 μm. **D.** Distribution of m^6^A sites along the 5′ UTR, CDS, and 3′ UTR regions of mRNAs from ND and HFD mouse livers. **E**. UPLC-MRM-MS/MS showing the percentage of m^6^A/A in mRNAs of ND and HFD mouse livers. *n* = 4. **F.** Distribution of genes with significant changes in both m^6^A level (hyper- or hypo-) and gene expression (up- or down-) under HFD condition. **G.** Significantly enriched (*P* < 0.05) GO (biological process) categories of genes with down-regulated expression and higher m^6^A level in HFD mouse liver. HFD mice were fed with a 60 kcal% fat diet for 20 weeks. Data are presented as mean ± SEM. Significant difference was determined by unpaired student’s *t*-test (*, *P* < 0.05; n.s., no significance). ND, normal diet; HFD, high fat diet; UPLC–MRM–MS/MS, ultra-performance liquid chromatography–triple quadrupole mass spectrometry coupled with multiple-reaction monitoring; m^6^A, *N*^6^-methyladenosine; GO, gene ontology. Raw data are displayed in Table S2.

To explore the potential role of m^6^A in the regulation of lipid and glucose metabolism of HFD-induced obese mice, we first measured the relative mRNA levels of m^6^A ‘writers’, ‘erasers’, and ‘readers’, including *Mettl3*, *Mettl14*, *Wtap*, *Fto*, *Alkbh5*, *Ythdf1*, *Ythdf2*, *Ythdf3*, *Ythdc1* (YTH domain-containing protein 1), and *Ythdc2*, in mouse liver after HFD (60 kcal% fat diet) for 20 weeks. The mRNA expression of m^6^A methyltransferases significantly increased in HFD mouse liver, while there was no difference in demethylases or m^6^A binding proteins (Figure 1A). Given Mettl3 is the key ‘writer’ of m^6^A modification [2], [3], we further confirmed the significantly increased protein level of Mettl3 by Western blotting and immunohistochemistry assay (Figure 1B and C).

To further investigate the underlying mechanisms of Mettl3-mediated m^6^A methylation in HFD-induced metabolic disorder, we performed RNA sequencing (RNA-seq) and m^6^A individual-nucleotide-resolution cross-linking and immunoprecipitation sequencing (miCLIP-seq) using mRNAs extracted from the livers of normal diet (ND) and HFD mice (Figure S1A). Consistent with previous reports [6], [27], the m^6^A sites on liver mRNAs were also enriched in the regions with RRACH motif (Figure S1B) and tended to occur near stop codons within CDS and in 3′ UTRs (Figure S1C). More importantly, we detected increased m^6^A sites in HFD mouse liver (Figure 1D). To further validate the presence of m^6^A modifications on the mRNAs of HFD mouse liver, we applied ultra-performance liquid chromatography–triple quadrupole mass spectrometry coupled with multiple-reaction monitoring (UPLC–MRM–MS/MS) analysis to quantify the m^6^A contents on mRNAs, and observed increased mRNA m^6^A modifications in HFD mouse liver (Figure 1E), which was consistent with the higher expression of Mettl3. In addition, 16,686 m^6^A sites were newly induced on mRNAs of HFD mouse liver corresponding to 1860 methylated genes (Figure S1D and Table S1). The proportion of unique m^6^A sites and overlapping m^6^A sites with higher level in HFD mouse liver also confirmed the increased m^6^A level on HFD mouse liver mRNAs (Figure S1E). To investigate the association of m^6^A with gene expression, we analyzed the RNA-seq data from ND and HFD mouse liver samples and identified 1913 differentially expressed mRNAs in total with 714 up-regulated genes and 1199 down-regulated genes (RPKM > 1). Meanwhile, we combined the gene expression data with m^6^A levels, and discovered 514 genes with increased m^6^A level in HFD mouse liver. Since it has been reported that the presence of m^6^A sites facilitated mRNA degradation [6], we mainly focused on the 409 genes with both hyper m^6^A level and down-regulated expression in HFD mouse liver (Figure 1F), as this group of transcripts was likely to be stabilized after m^6^A depletion. Gene Ontology (GO) analysis revealed that most of these genes were enriched in lipid metabolic processes, including acylglycerol metabolic process and fatty acid metabolic process. Glycometabolism related pathways, such as regulation of carbohydrate catabolic process, were also enriched (Figure 1G). Taken together, m^6^A level and its methyltransferase Mettl3 were consistently up-regulated in the liver of HFD mice, indicating that Mettl3-mediated m^6^A methylation might be involved in metabolic disorders induced by HFD.

### Overexpression of ***Mettl3*** aggravated liver metabolic disorders and hepatogenous diabetes

To confirm the relationship between high expression level of *Mettl3* and HFD-induced metabolic disorders, we specifically overexpressed *Mettl3* in mouse liver by hepatocyte-targeted AAV8 [28] and hepatocyte-specific promoter (*LP1*) [29] (Figure S2A). Living imaging reconfirmed the specifically expressed luciferase in mouse liver at 4 weeks after AAV retro orbital injection, which demonstrated that *Mettl3* was also specifically expressed in liver (Figure S2B). Moreover, qRT-PCR and Western blotting revealed the successful overexpression of *Mettl3* in liver (Figure S2C and D).

**Figure 2:**
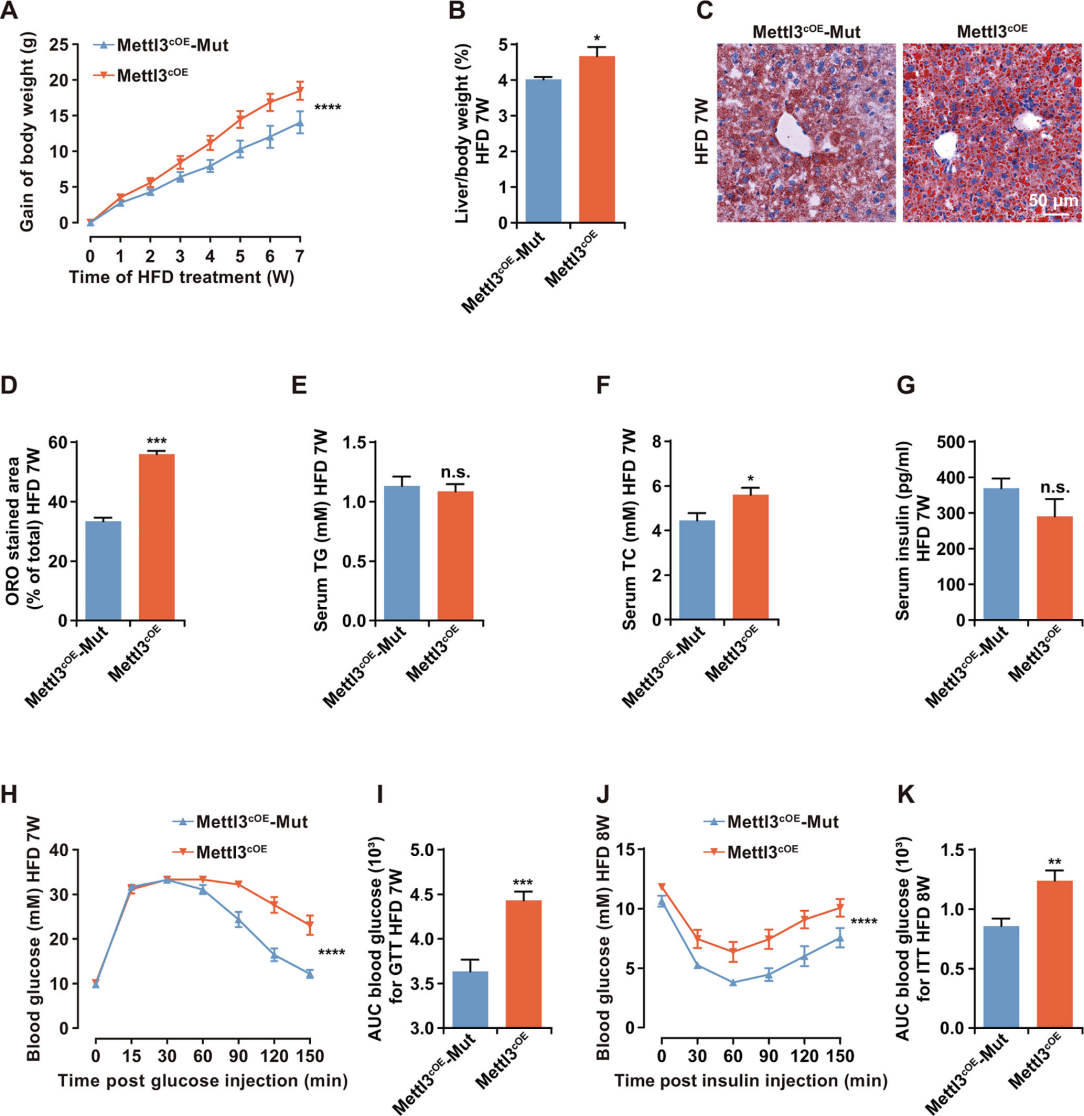
**Overexpression of *Mettl3* aggravated liver metabolic disorders and hepatogenous diabetes** **A.** Body weight gain curve of Mettl3^cOE^-Mut and Mettl3^cOE^ mice during 7 weeks of HFD treatment. Gain of body weight (g) = final body weight (g) – initial body weight (g). *n* = 10. **B.** The ratio of liver weight to body weight for Mettl3^cOE^-Mut and Mettl3^cOE^ mice after 7 weeks of HFD treatment. *n* = 5. **C.** Representative photomicrographs of ORO stained livers of Mettl3^cOE^-Mut and Mettl3^cOE^ mice after 7 weeks of HFD treatment. Scale bar, 50 μm. **D.** The proportion of ORO stained area in Mettl3^cOE^-Mut and Mettl3^cOE^ mouse livers after 7 weeks of HFD treatment. *n* = 3. **E**.**–G.** Serum TG (E), TC (F), and insulin (G) contents of Mettl3^cOE^-Mut and Mettl3^cOE^ mice after 7 weeks of HFD treatment. *n* = 10. **H.** Blood glucose curve of Mettl3^cOE^-Mut and Mettl3^cOE^ mice after 7 weeks of HFD treatment during GTT. *n* = 8. **I.** AUC statistics for (H). *n* = 8. **J.** Blood glucose curve of Mettl3^cOE^-Mut and Mettl3^cOE^ mice after 8 weeks of HFD treatment during ITT. *n* = 8. **K.** AUC statistics for (J). *n* = 8. Data are presented as mean ± SEM. Significant difference was determined by unpaired student’s *t*-test (*, *P* < 0.05; **, *P* < 0.01; ***, *P* < 0.001; ****, *P* < 0.0001; n.s., no significance). Mettl3^cOE^-Mut, mutant *Mettl3* (DPPW → APPA) conditional overexpression mice, served as a control; Mettl3^cOE^, *Mettl3* conditional overexpression mice; ORO, Oil Red O; TG, triglyceride; TC, total cholesterol; GTT, glucose tolerate test; ITT, insulin tolerate test; AUC, area under the curve. Raw data are displayed in Table S2.

We tracked the changes in mouse body weight and metabolic parameters in response to HFD. Compared with mutant *Mettl3* conditional overexpression mice (Mettl3^cOE^-Mut, served as a control), *Mettl3* conditional overexpression mice (Mettl3^cOE^) showed more increase in body weight during HFD (Figure 2A), due to more subcutaneous fat in Mettl3^cOE^ mice (Figure S2E). The ratio of liver weight to body weight, as well as Oil Red O (ORO) staining, further revealed that Mettl3^cOE^ mice presented more serious hepatic steatosis (Figure 2B–D). Moreover, compared with Mettl3^cOE^-Mut mice, serum total cholesterol (TC) of Mettl3^cOE^ mice also increased, while there was no significant change in total triglyceride (TG) (Figure 2E and F).

Although there was no significant change in serum insulin level (Figure 2G), glucose tolerance test (GTT) showed that Mettl3^cOE^ mice presented significantly worse glucose tolerance than Mettl3^cOE^-Mut mice in HFD condition (Figure 2H and I). Besides, insulin tolerance test (ITT) also revealed that insulin sensitivity of Mettl3^cOE^ mice was also notably worse than Mettl3^cOE^-Mut mice in HFD condition (Figure 2J and K). Together, these results indicate that *Mettl3* overexpression can aggravate liver metabolic disorders and hepatogenous diabetes, suggesting that high level of Mettl3 may be a risk factor for HFD-induced metabolic syndrome.

### ***Mettl3*** ablation protected mice against HFD-induced metabolic syndrome

Considering that overexpression of *Mettl3* can aggravate liver metabolic disorders and hepatogenous diabetes induced by HFD, we supposed that *Mettl3* ablation in liver could resist HFD-induced metabolic syndrome. To verify this hypothesis, we generated *Mettl3* conditional knockout mice (Mettl3^cKO^) by crossing *Alb-*Cre and *Mettl3*^flox/flox^ mice (Figure S3A). Cre enzyme were specifically expressed in liver and produced *Mettl3* transcripts without exons 2–4. Moreover, Cre enzyme didn’t leak into other tissues (Figure S3B). qRT-PCR, Western blotting, and immunohistochemistry assay together confirmed the successful deletion of *Mettl3* in liver at both mRNA and protein levels (Figure S3C–E).

**Figure 3:**
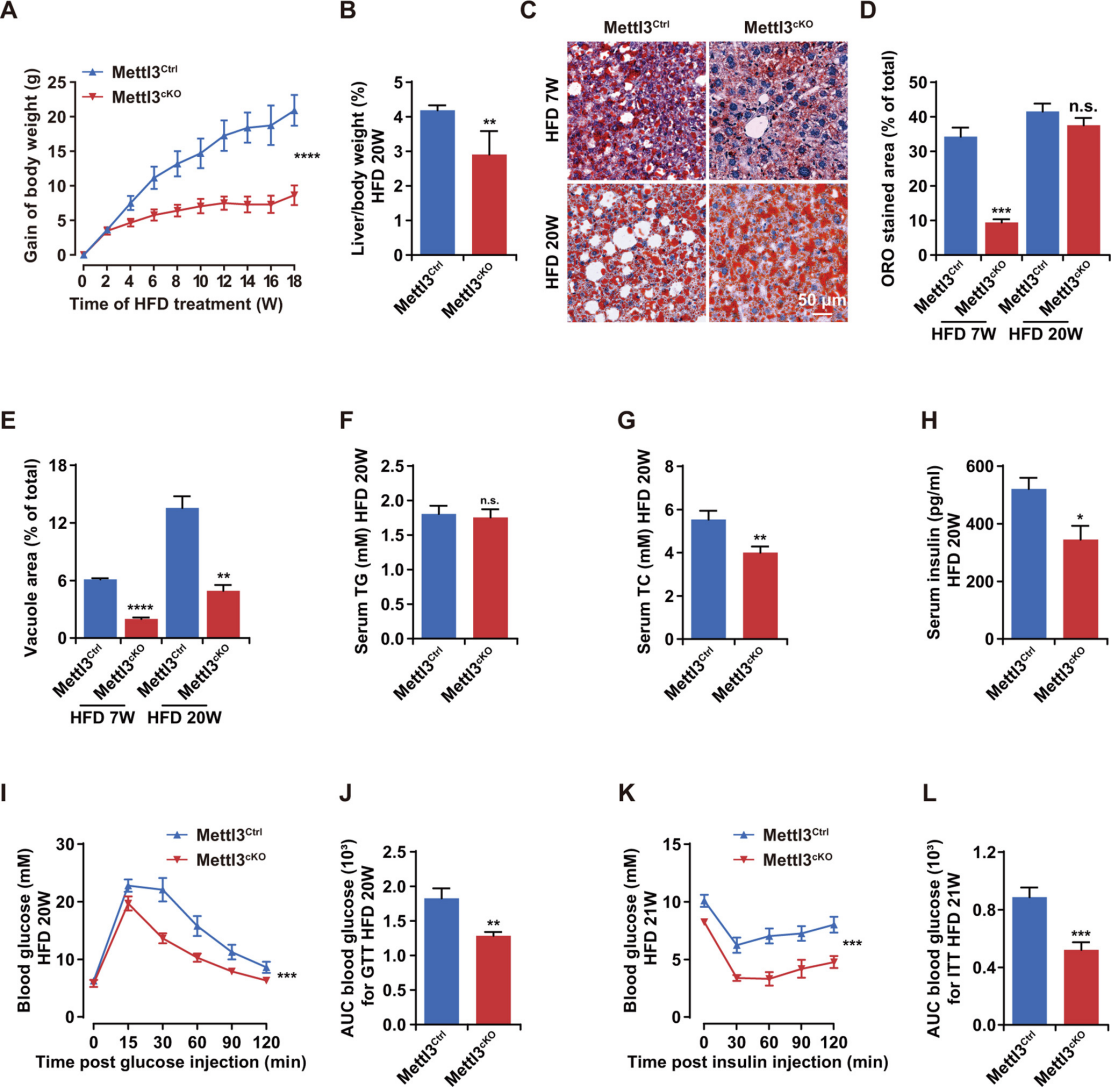
***Mettl3* ablation protected mice againstHFD-induced metabolic syndrome** **A.** Body weight gain curve of Mettl3^Ctrl^ and Mettl3^cKO^ mice during 20 weeks of HFD treatment. *n* = 10. **B.** The ratio of liver weight to body weight for Mettl3^Ctrl^ and Mettl3^cKO^ mice after 20 weeks of HFD treatment. *n* = 5. **C.** Representative photomicrographs of ORO stained livers of Mettl3^Ctrl^ and Mettl3^cKO^ mice after 7 weeks and 20 weeks of HFD treatment. Scale bar, 50 μm. **D.** The proportion of ORO stained area in Mettl3^Ctrl^ and Mettl3^cKO^ mouse livers after 7 weeks and 20 weeks of HFD treatment. *n* = 3. **E.** The proportion of vacuole area in Mettl3^Ctrl^ and Mettl3^cKO^ mouse livers after 7 weeks and 20 weeks of HFD treatment. *n* = 3. **F.**–**H.** Serum TG (F), TC (G), and insulin (H) contents of Mettl3^Ctrl^ and Mettl3^cKO^ mice after 20 weeks of HFD treatment. *n* = 8. **I.** Blood glucose curve of Mettl3^Ctrl^ and Mettl3^cKO^ mice after 20 weeks of HFD treatment during GTT. *n* = 10. **J.** AUC statistics for (I). *n* = 10. **K.** Blood glucose curve of Mettl3^Ctrl^ and Mettl3^cKO^ mice after 21 weeks of HFD treatment during ITT. *n* = 10. **L.** AUC statistics for (K). *n* = 10. Data are presented as mean ± SEM. Significant difference was determined by unpaired student’s *t*-test (*, *P* < 0.05; **, *P* < 0.01; ***, *P* < 0.001; ****, *P* < 0.0001; n.s., no significance). Mettl3^Ctrl^, *Mettl3*^flox/flox^ mice; Mettl3^cKO^, *Mettl3*^flox/flox^;*Alb-*Cre mice. Raw data are displayed in Table S2.

As expected, the body weight of Mettl3^cKO^ mice increased more slowly than Mettl3^Ctrl^ (Figure 3A), and they also had less subcutaneous fat than Mettl3^Ctrl^ after HFD for 20 weeks (Figure S3F). HFD-induced hepatic steatosis was slighter in Mettl3^cKO^ mouse liver, which was evaluated by the ratio of liver weight to body weight and ORO staining (Figure 3B–D). However, in the late stage of HFD, Mettl3 depletion seemed could not confront the lipid accumulation significantly. We speculated that in the late stage of HFD, lipid accumulation may have reached the limit of liver. However, more numerous and larger vacuoles in Mettl3^Ctrl^ mouse liver indicated that the damage of Mettl3^Ctrl^ mouse liver was more serious than Mettl3^cKO^ (Figure 3E). In addition, although there was no significant change in serum TG, serum TC of Mettl3^cKO^ mice decreased in HFD condition, consistent with the phenotype of Mettl3^cOE^ mice (Figure 3F and G).

It’s worth noting that serum insulin level significantly decreased in Mettl3^cKO^ mice in HFD condition (Figure 3H). Meanwhile, consistent with the glucometabolic phenotype of Mettl3^cOE^ mice, Mettl3^cKO^ mice presented significantly better glucose tolerance (Figure 3I and J) and insulin sensitivity (Figure 3K and L) than Mettl3^Ctrl^ in HFD condition. Taken together, these results suggest that Mettl3 depletion in liver could protect mice against HFD-induced metabolic syndrome, indicating that Mettl3 might be a potential therapeutic target for liver metabolic diseases.

### ***Mettl3*** ablation altered the expression pattern of lipid and glucose metabolic genes

**Figure 4:**
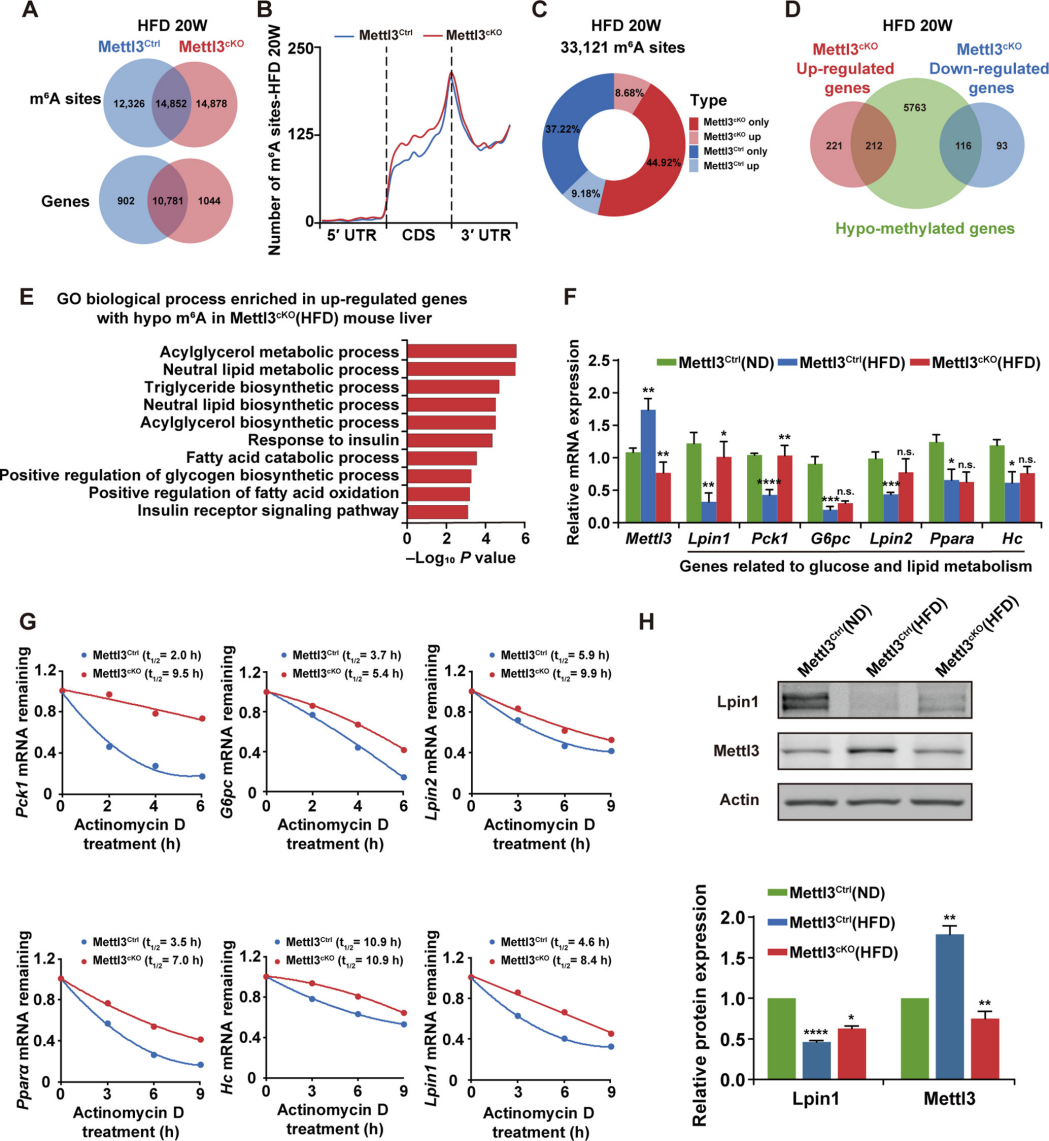
***Mettl3* ablation altered the expression pattern of lipid and glucose metabolic genes** **A.** Venn diagram depicting the number of unique and overlapping m^6^A sites on liver mRNAs from Mettl3^Ctrl^(HFD) and Mettl3^cKO^(HFD) mice, as well as the number of unique and overlapping methylated genes in Mettl3^Ctrl^(HFD) and Mettl3^cKO^(HFD) mouse livers. Numbers represent the counts of m^6^A sites or methylated genes in each group. **B.** Distribution of m^6^A sites along the 5ʹ UTR, CDS, and 3ʹ UTR regions of liver mRNAs from Mettl3^Ctrl^(HFD) and Mettl3^cKO^(HFD) mice. **C.** Donut chart showing the proportion of unique m^6^A sites (only) and overlapping m^6^A sites with higher level (up) in the livers of Mettl3^Ctrl^(HFD) and Mettl3^cKO^(HFD) mice. **D.** Venn diagram representing the relationships between altered genes (up-regulated or down-regulated in Mettl3^cKO^(HFD) mice liver) and lower m^6^A level. Numbers represent the counts of genes in each group. **E.** GO (biological process) categories (*P* < 0.05) of genes with up-regulated expression and lower m^6^A level in the livers of Mettl3^cKO^(HFD) mice. **F.** qRT-PCR validation of liver lipid and glucose metabolic genes. All of these genes were down-regulated in Mettl3^Ctrl^(HFD) mouse liver (compare with Mettl3^Ctrl^(ND)), while only *Lpin1* and *Pck1* were up-regulated in Mettl3^cKO^(HFD) mouse liver (compare with Mettl3^Ctrl^(HFD)). *Ubc* served as the internal control. *n* = 5. **G.** mRNA half-lives of *Pck1*, *G6pc*, *Lpin2*, *Pparα*, *Hc*, and *Lpin1*. mRNA levels were measured by qRT-PCR at the indicated time points after Actinomycin D treatment. *Ubc* served as the internal control. *n* = 3. **H.** Western blotting detection and quantification of Lpin1 and Mettl3 protein expression in liver extracts from Mettl3^Ctrl^(ND), Mettl3^Ctrl^(HFD), and Mettl3^cKO^(HFD) mice. Actin served as the loading control. *n* = 3. All mouse liver samples were prepared after 20 weeks of HFD treatment. Data are presented as mean ± SEM. Significant difference was determined by unpaired student’s *t*-test (*, *P* < 0.05; **, *P* < 0.01; ***, *P* < 0.001; ****, *P* < 0.0001; n.s., no significance). Raw data were displayed in Table S2.

To further explore the underlying mechanisms of Mettl3 depletion in protecting liver from metabolic syndrome induced by HFD, we analyzed RNA-seq and miCLIP-seq data generated from livers of Mettl3^Ctrl^ and Mettl3^cKO^ mice after 20 weeks of HFD (termed as Mettl3^Ctrl^(HFD) and Mettl3^cKO^(HFD), respectively). Similarly, the m^6^A sites on Mettl3^cKO^(HFD) mouse liver mRNAs were enriched in the regions with RRACH motif (Figure S4A), and tended to occur near stop codons within CDS and in 3′ UTRs of mRNAs (Figure S4B). Within all the methylated mRNAs, around 35.1% of methylated mRNAs were found to contain one m^6^A site (Figure S4C and Table S1). Since HFD-induced *Mettl3* up-regulation and *Mettl3* knockout genetic manipulation had opposite effects on m^6^A level, there was no obvious difference in the number of m^6^A sites or methylated genes between Mettl3^Ctrl^(HFD) and Mettl3^cKO^(HFD) mouse livers (Figure 4A). Meanwhile, the m^6^A sites across the entire gene bodies of Mettl3^Ctrl^(HFD) and Mettl3^cKO^(HFD) mouse livers also displayed similar distribution (Figure 4B). However, it seemed that HFD-induced Mettl3 up-regulation played a more dominant role, because the proportion of unique m^6^A sites and overlapping m^6^A sites with higher level in Mettl3^cKO^ mouse liver was greater than that in Mettl3^Ctrl^ in HFD condition (Figure 4C). Among the hypo-methylated genes, 212 genes were up-regulated while 116 genes were down-regulated in Mettl3^cKO^(HFD) mouse liver (Figure 4D). Given that m^6^A was mainly reported to play a negative role in mRNA stability regulation, we focused on the m^6^A-containing up-regulated genes in Mettl3^cKO^(HFD) mouse liver, performed GO analysis, and found that these genes were enriched in insulin response and lipid metabolic related processes (Figure 4E).

qRT-PCR further validated the expression of these candidate genes which were down-regulated in Mettl3^Ctrl^(HFD) mouse liver (compared with Mettl3^Ctrl^(ND)), such as *Lpin1*, *Pck1*, *G6pc*, *Lpin2*, *Pparα*, and *Hc*. Among them, *Lpin1* and *Pck1* were up-regulated in Mettl3^cKO^(HFD) mouse liver (compared with Mettl3^Ctrl^(HFD)) (Figure 4F). mRNA stability assay revealed that most of these candidate genes were more stable in Mettl3^cKO^ mouse liver due to Mettl3 depletion-induced m^6^A loss (Figure 4G). Among them, *Lpin1* has been reported to play an important role in liver lipid metabolism and insulin resistance [30], [31], [32], [33], Furthermore, Lpin1 protein decreased in Mettl3^Ctrl^(HFD) mouse liver (compared with Mettl3^Ctrl^(ND)) while increased in Mettl3^cKO^(HFD) mouse liver (compared with Mettl3^Ctrl^(HFD)). Its expression pattern was contrary to that of Mettl3 protein (Figure 4H). Collectively, these findings demonstrate that *Mettl3* ablation stabilized key lipid and glucose metabolic genes, especially improved the stability of *Lpin1* mRNA through modulating m^6^A levels.

## Discussion

As the most prevalent mRNA modification in eukaryotes [1], m^6^A involves in many essential biological processes, including cell fate determination [12], [13], embryonic development [13], [14], [15], and tumorigenesis [16]. Recent studies have demonstrated that m^6^A modulation of mRNA expression plays important roles in adipogenesis [18], hepatic lipid metabolism [20], obesity [21], and other metabolic diseases, such as NAFLD and type 2 diabetes (T2D) [21], [22], [23], [24], [25], [26]. However, the underlying mechanisms and pathways by which Mettl3-mediated m^6^A modification regulates liver metabolism remain unclear.

A recent study reported that Mettl3 inhibited hepatic insulin sensitivity via m^6^A located in *Fasn* mRNA and promoted fatty acid metabolism [26]. In our current work, we present several findings demonstrating the significance of m^6^A in HFD-induced liver metabolic disorders: 1) The major m^6^A methyltransferase Mettl3 and m^6^A level were consistently elevated in the liver of mouse after feeding HFD. 2) AAV8-mediated liver conditional overexpression of *Mettl3* aggravated liver and whole-body metabolic disorders, including liver lipid accumulation, abnormal serum TC and obesity. Moreover, Mettl3^cOE^ mice presented worse glucose tolerance and insulin sensitivity compared with Mettl3^cOE^-Mut mice (served as a control). 3) Mettl3^cKO^ mouse model generated by crossing *Alb-*Cre and *Mettl3*^flox/flox^ mice confirmed that *Mettl3* ablation protected mice against HFD-induced liver metabolic disorders and hepatogenous diabetes. 4) *Mettl3* ablation stabilized key genes involved in liver lipid and glucose metabolism, and particularly elevated the mRNA stability of an important regulator of hepatic lipid and glucose metabolism, *Lpin1*. Collectively, our findings demonstrate the critical roles for Mettl3-mediated m^6^A modification in HFD-induced liver metabolic disorders and hepatogenous diabetes, supporting that m^6^A might be a potential therapeutic and diagnostic target for hepatic diseases.

Previous studies have shown that *METTL3* was elevated in peripheral venous blood and livers of T2D patients [26], [34], while the reason for *METTL3* increase was considered as a result of FTO-induced decrease in m^6^A. High-glucose stimulation elevated *FTO* expression, which led m^6^A to decrease, as a response, *METTL3* might increase to maintain the normal m^6^A level [34]. In the present study, we also detected elevated *Mettl3* in the liver of mice after feeding HFD; however, the expression of *Fto* didn’t show a significant increase. Therefore, we highly speculate that the up-regulation of *Mettl3* in HFD mouse liver may result from other signal pathways.

Several studies showed that Fto-mediated m^6^A demethylation positively regulated adipogenesis. For instance, m^6^A demethylation promoted adipogenesis in porcine intramuscular preadipocytes through inhibiting the Wnt/β-catenin signal pathway [35], increased adipogenesis in mouse embryonic fibroblasts and primary preadipocytes by regulating mitotic clonal expansion [36], and controlled adipogenesis through the regulation of cell cycle in an Ythdf2-m^6^A-dependent manner [37]. Moreover, consistent with these *in vitro* studies, *Fto* overexpression induced adipocyte hyperplasia in HFD mice [36]. Conversely, Mettl3 negatively correlated with adipogenesis in porcine adipocytes through m^6^A methylation [38], which seems to conflict with the weight loss of Mettl3^cKO^ mice after HFD as we observed. However, obesity is caused by many factors. In this study, it was the result of initial liver metabolic disorders and hepatogenous diabetes, rather than adipogenesis or proliferation of preadipocytes. Meanwhile, the lipid accumulation in hepatocytes was a comprehensive result of liver lipid synthesis, catabiosis, and transportation. *Mettl3* was likely to be involved in all these processes and eventually increased lipid accumulation in HFD mouse liver.

It is interesting to note that lipid accumulation unexpectedly increased in Mettl3^cKO^ mouse liver in ND condition (data not shown), contrary to the corresponding phenotype in HFD condition. By high- throughput RNA-seq and miCLIP-seq, we compared the hypo-methylated up-regulated genes in Mettl3^cKO^ mouse liver under both ND and HFD conditions. We found that Mettl3-targeted genes were enriched in sterol biosynthetic process in ND condition, while in several catabolism pathways in HFD condition, such as fatty acid catabolic process and positive regulation of fatty acid oxidation. These findings indicate that Mettl3 might regulate different subsets of genes in different diet conditions and serve as a bidirectional switch in lipid metabolism.

Taken together, we found that Mettl3 served as an essential regulator of liver lipid and glucose metabolism. It could protect mice from metabolic disorders and hepatogenous diabetes induced by HFD. These results will promote Mettl3-mediated m^6^A as a target for hepatic diseases’ therapy and diagnosis.

## Materials and methods

### Mice

The mice used in this study were C57BL/6 strains. Specific pathogen-free-grade mice were purchased from Beijing Charles River Laboratory Animal Center and housed in the animal facilities of the Institute of Zoology, Chinese Academy of Sciences (CAS), China.

### Mouse breeding

*Mettl3*^flox/+^ mice were generated by the CRISPR-Cas9 system-assisted homologous recombination as previously described [39]. C57BL/6 *Alb-*Cre transgenic mice were purchased from Shanghai BRL Medicine Company (China). *Mettl3*^flox/flox^ mice were obtained by mating *Mettl3*^flox/+^ to each other. *Mettl3*^flox/+^;*Alb*-Cre mice were obtained by mating *Mettl3*^flox/flox^ and *Alb*-Cre mice. *Mettl3*^flox/+^;*Alb*-Cre and *Mettl3*^flox/flox^ mice were crossed to generate *Mettl3*^flox/flox^;*Alb*-Cre (Mettl3^cKO^) mice.

### Genotyping of mice

All mice were genotyped with the tail DNA which was extracted using the Mouse Direct PCR Kit (Catalog No. B40015, Bimake, Houston, TX). Briefly, mouse tails were mixed with 50 μl Buffer L and 1 μl Protease Plus, incubated at 55 °C for 30 min, and then incubated at 100 °C for 5 min according to the manufacturer’s instructions.

Two pairs of primers were used to detect the loxp insertions into the *Mettl3* intron 1 (L-loxp-F and L-loxp-R) and intron 4 (R-loxp-F and R-loxp-R). The product sizes were 222 bp and 335 bp for the loxp sequence insertions into *Mettl3* intron 1 and intron 4, respectively; whereas the product sizes for WT were 182 bp and 295 bp, respectively. Cre recombinase was detected by the *Alb-*Cre primers, and its PCR product was 350 bp. Heart, liver, spleen, lung, kidney, and brain were detected to confirm the deletion of *Mettl3* with the primers L-loxp-F and R-loxp-R. The *Mettl3* deletion product was 318 bp, whereas the WT product was 2554 bp. All primers are listed in Table S3.

### RNA extraction and qRT-PCR

Total RNA was extracted from the whole liver with TRIzol Reagent (Catalog No. 15596-018, Invitrogen, Carlsbad, CA), and then reverse-transcribed into cDNA using the Reverse Transcription System (Catalog No. A3500, Promega, Madison, WI). qRT-PCR was performed using SYBR Premix Ex Taq kit (Catalog No. RR420A, TaKaRa, Kyoto, Japan) on Agilent Stratagene Mx3005P. Relative gene expression was analyzed based on the 2^−ΔΔCt^ method with *Ubc* as the internal control. All primers are listed in Table S3.

### Western blotting

Western blotting was performed as described previously [40] with corresponding antibodies: anti-Mettl3 (1:500; catalog No. ab195352, Abcam, Cambridge, UK), anti-Lpin1 (1:500; catalog No. 5195S, Cell Signalling Technology, Bossdun, MA), anti-β-Actin (1:2000; catalog No. A1978, Sigma, St. Louis, MO), and anti-α-Tubulin (1:2000; catalog No. T6199, Sigma).

### Immunohistochemistry assay

Immunohistochemistry was performed as described previously [40]. Anti-Mettl3 and Hoechst 33342 (1:1000; catalog No. H3570, Invitrogen) were used. Images were obtained using standard methods with a Leica Aperio VERSA 8 microscope (Leica Biosystems, Wetzlar, Germany).

### Plasmid construction and virus production

pX602 backbone was modified from pX602-AAV-TBG::NLS-SaCas9-NLS-HA-OLLAS-bGHpA;U6::BsaI-sgRNA, which was a gift from Feng Zhang (Addgene plasmid #61593; http://n2t.net/addgene:61593; RRID: Addgene_61593) [41]. *LP1* promoter was constructed as previously described [29]. Mettl3 catalytic mutant (395–398 aa, DPPW → APPA) was also generated as previous work [42].

AAV8 was generated with HEK-293 cells, purified with chloroform, titered by qPCR as previously described [43], and then retro orbital injected into mice at the titer of 2.5 × 10^12^ vg each mouse.

### Oil Red O staining

Liver lipid accumulation was confirmed by Modified Oil Red O stain kit (Catalog No. G1261, Solarbio, Beijing, China) according to the manufacturer’s instructions. In brief, frozen slices of liver (6–10 μm) were fixed in 10% formaldehyde for 10 min, and then washed with 60% isopropanol for 20–30 s. Liver tissue was stained in Modified Oil Red O solution for 10–15 min. After staining, the slices were washed with 60% isopropanol and then with H_2_O. Images were obtained using standard methods, imaged with a Leica Aperio VERSA 8 microscope, and then analyzed with Image J (1.48v, Bethesda, MD).

### Metabolic measurements

For GTT assay, mice were fasted overnight (for 12 h) and then injected intraperitoneally (i.p.) with D-glucose (2 g/kg body weight; catalog No. G8270, Sigma). For ITT assay, mice were randomly fed and injected i.p. with insulin from porcine pancreas (0.75 U/kg body weight; catalog No. I113907, Aladdin, Shanghai, China). Blood from a tail vein was collected before injection and at different time points after injection (as indicated in the figures). Glucose concentrations were measured with AccuCheck blood glucose meter (Roche Diagnostics Inc., Basel, Switzerland). Serum TG and TC concentrations were measured with Automatic biochemical analyzer (Catalog No. Chemray 240, Shenzhen, China). Serum insulin concentrations were measured by the Insulin test ELISA kit (Catalog No. CEA448Mu, USCN KIT INC., Wuhan, China), and performed as manufacturer’s instructions.

### Fat volume measurements

Mice were anesthetized with isoflurane, put into the Quantum FX system (PE Quantum FX, PerkinElmer, Waltham, MA), and then scanned with X-ray. Data are analyzed with Analyze 12.0 (AnalyzeDirect, Overland Park, KS).

### UPLC–MRM–MS/MS analysis

mRNAs were purified from total RNAs using Dynabeads mRNA purification kit (Catalog No. 61006, Ambion, Carlsbad, CA). 200 ng mRNA was mixed with 0.1 U Nuclease P1 from *Penicillium citrinum* (Catalog No. N8630, Sigma) and 2.0 U Alkaline Phosphatase, Calf Intestinal (Catalog No. M0290L, New England Biolabs, Ipswich, UK). The final reaction volume was 40 μl. The mixture was incubated at 37 °C overnight, and then transferred to ultrafiltration tubes (MW cutoff of 3 kDa; catalog No. OD003C35, Pall, New York, NY) and centrifuged at 14,000 *g* at 4 °C for 25 min.

The UPLC–MRM–MS/MS analysis was performed according to a previous report [44]. The LC was performed on an ExionLCTM analytical system (Sciex, Framingham, MA). Chromatographic separation was carried out on an Acquity UPLC HSS T3 column (1.8 μm, 100 mm × 2.1 mm ID; catalog No. 186003539, Waters, Milford, MA). The flow rate was 0.25 ml/min. The mobile phase consisted of methanol (solvent A) and water containing 0.1% formic acid (solvent B) in a linear gradient. The gradient program was as follows: 0–2.5 min, 4% A; 2.5–2.7 min, 4%–31% A; 2.7–6 min, 31% A; 6–6.2 min, 31%–95% A; 6.2–9.3 min, 95% A; 9.3–9.6 min, 95%–4% A; 9.6–14.5 min, 4% A. The column temperature was maintained at 40 °C. The temperature of the autosampler was set at 4 °C, and the injection volume was 4 μl.

MS/MS analysis was carried out on a Qtrap 4500 mass spectrometer (Sciex, Framingham, MA) equipped with Turbo Ion spray interface operating in positive ESI mode. The instrument was operated with an ion spray voltage of 4.5 kV and a heater gas temperature of 500 °C. A nebulizer gas (gas 1) of 40 psi, a heater gas (gas 2) of 50 psi, a curtain gas of 20 psi, and a medium collision gas were used. Mass-dependent parameters, such as the declustering potential, entrance potential, collision energy, and collision cell exit potential, were set to the optimal values obtained by automated optimization. A multiple reaction monitoring (MRM) mode was employed for data acquisition. *m*/*z* 282.1 → 150.1 was for m^6^A (collision energy, 12 eV), and *m*/*z* 268.1 → 136.1 was for A (9 eV). The injection volume for each sample was 5 μl. The amounts of m^6^A and A were calibrated by standard curves. The dwell time for each transition was 100 ms. Data acquisition was performed with Analyst 1.6.2 software (Applied Biosystems, Waltham, MA).

### mRNA stability assay

Primary hepatocytes were plated on 6-well plates with 5 × 10^5^ cells per well and cultured for 2 days. Then cells were treated with actinomycin-D (10 μg/ml; catalog No. HY-17559, MCE, Monmouth Junction, NJ) and collected at the indicated time points (2, 4, and 6 h or 3, 6, and 9 h). Total RNA was extracted and analyzed by qRT-PCR. *Ubc* was used as the internal control. The half-life of gene was calculated as previously described [40]. Three replicates were conducted for each calculation.

### RNA-seq and m^6^A-miCLIP-seq

RNA-seq libraries were directly generated using the KAPA Stranded mRNA-Seq Kit (Catalog No. KK8401, KAPA Biosystem, Bossdun, MA) following the manufacturer’s instructions.

The preparation of miCLIP-seq libraries was carried out following previously reported methods [45], [46] with some modifications. Briefly, mRNAs purified using Dynabeads mRNA Purification Kit (Catalog No. 61006, Ambion) were fragmented to a size of around 100 nt with the fragmentation reagent (Catalog No. AM8740, Life Technologies, New York, NY). Then, 2 μg purified mRNAs were mixed with 5 μg anti-m^6^A antibody (Catalog No. ab151230, Abcam) in 450 μl immunoprecipitation buffer (50 mM Tris, pH 7.4, 100 mM NaCl, and 0.05% NP-40), and incubated by rotating at 4 °C for 2 h. The solution was then transferred to a clear flat-bottom 96-well plate on ice and irradiated three times with 0.15 J/cm^2^ at 254 nm in a CL-1000 Ultraviolet Crosslinker (UVP). The mixture was then immunoprecipitated through incubation with Dynabeads Protein A (Catalog No. 1001D, Life Technologies) at 4 °C for 2 h. After extensive washing, on-bead end-repair, and linker ligation, the bound RNA fragments were eluted from the beads by proteinase K digestion at 55 °C for 1 h. RNAs were isolated by further phenol–chloroform extraction and ethanol precipitation. Purified RNAs were used to construct the library using SMARTer smRNA-Seq Kit for Illumina (Catalog No. 635029, Takara) according to the manufacturer’s instructions. Sequencing was carried out on Illumina HiSeq X-ten platform with paired-end 150-bp read length.

### Analysis of RNA-seq data

All the RNA-seq samples were sequenced by Illumine Hiseq X ten platform with paired-end 150-bp read length. Clean fastq reads after quality control by cutadapt and Trimmomatic [48] were aligned to mouse reference genome (GRCm38/mm10; Ensembl version 68) via HISAT2 (v2.0.5) aligner [47] with default settings. Only the reads with mapping quality score (MAPQ) ≥ 20 were kept for the downstream analysis. FeatureCounts (v1.6.0) [49] was employed to estimate the read counts per gene according to library type. Differentially expressed genes were identified by edgeR (v3.18.1) [50] with fold change (FC) > 1.5 and *P* < 0.05 as thresholds between ND and HFD or between Mettl3^Ctrl^(HFD) and Mettl3^cKO^(HFD) groups. In the whole process, we only kept the genes with reads per kilobase per million mapped reads (RPKM) > 1 as the candidate genes for further analysis. GO (biological process) enrichment analysis (*P* < 0.05) was performed using the R package clusterProfiler [51].

### Analysis of miCLIP-seq data

#### Read processing

Raw sequencing data quality control was performed by FastQC. Adaptors were trimmed by fastx_clipper tool from FASTX-Toolkit (http://hannonlab.cshl.edu/fastx_toolkit). For the forward reads, PCR-amplified reads were removed by fastq2collapse.pl from CLIP Tool Kit (v1.0.3) [52] via barcode sequence. Cutadapt (v1.16) [48] was employed to trim the polyA-tail. Reverse reads were reversely complemented by fastx_reverse_complement tool from FASTX-Toolkit and processed in the same way. Random barcode removal was accomplished by stripBarcode.pl from CLIP Tool Kit (v1.0.3) [52], and only reads longer than 18 nt were kept by Trimmomatic (v0.33) [53].

#### Mapping and mutation calling

Replicate samples were merged and aligned to mouse reference genome (GRCm38/mm10; Ensembl version 68) by Burrows-Wheeler Alignment tool (v0.7.17-r1188) [54] with the recommend parameter, –n 0.06 –q 20. Cross-linking-induced mutation sites (CIMS) were detected by the CLIP Tool Kit (v1.0.3) [52] as reported. For each detected mutation site, the CIMS software identified the coverages of unique tags (k) and mutation position (m). In order to reduce false positive rates, we only kept the sites with an m/k ratio 1%–50% and mutation sites within the RRACH motif as reliable m^6^A sites for subsequent analysis [55]. m^6^A site annotation was performed by intersectBed from BEDTools (version 2.16.2) [56]. The m^6^A motif was generated by WebLogo3 [57]. For the differential m^6^A methylation sites, the read counts span per m^6^A site were calculated by the BEDTools multicov tool (version 2.16.2) [56] from miCLIP-seq and related RNA-seq data divided by the library size. The difference of m^6^A enrichment values between control and condition samples was determined by Chi-square test with *P* < 0.05. Meanwhile, we kept FC > 1.2 as threshold.

### Statistical analysis

All data are expressed as mean ± SEM. GraphPad Prism 8 (GraphPad Software Inc., San Diego, CA) was used for statistical analysis. Unpaired student’s *t*-test was used to determine the difference between two groups; a two-way ANOVA analysis followed by Bonferroni multiple-comparison test was used to determine differences among multiple groups. *P* < 0.05 was considered statistically significant.

## Ethical statement

All animal experiments were carried out under the guidelines for the use of animals in research issued by the Institute of Zoology, CAS, China, and were approved by the Institutional Animal Care and Use Committee.

## Data availability

The raw data for the RNA-seq and miCLIP-seq have been deposited in the Genome Sequence Archive [58] at the National Genomics Data Center, Beijing Institute of Genomics, Chinese Academy of Sciences / China National Center for Bioinformation (GSA: CRA002000), and are publicly accessible at http://bigd.big.ac.cn/gsa.

## CRediT author statement

**Yuhuan Li:** Methodology, Visualization, Writing - original draft. **Qingyang Zhang:** Formal analysis, Writing - original draft. **Guanshen Cui:** Methodology. **Fang Zhao:** Methodology. **Xin Tian:** Resources. **Bao-Fa Sun:** Formal analysis. **Ying Yang:** Conceptualization, Writing - original draft, Writing - review & editing, Supervision. **Wei Li:** Conceptualization, Writing - original draft, Writing - review & editing, Supervision. All authors read and approved the final manuscript.

## Competing Interests

The authors have declared no competing interests.

## Supplementary data

Supplementary Figure S1 **m^6^A pattern in HFD mouse liver**. **A**. Schematic illustration of miCLIP library construction procedure. **B**. m^6^A consensus motif in mRNAs from HFD mouse liver. **C**. Transcriptome-wide distribution of m^6^A sites. Pie chart shows the proportion of m6A sites in distinct non-overlapping segments: 5′ UTR, TSS, CDS, stop codon, and 3′ UTR. **D**. Venn diagram depicting the number of unique and overlapping m6A sites on liver mRNAs from ND and HFD mice, as well as the number of unique and overlapping methylated genes in ND and HFD mouse livers. Numbers represent the counts of m6A sites or methylated genes in each group. E. Donut chart showing the proportion of unique m6A sites (only) and overlapping m6A sites with higher level (up) in the livers of ND and HFD mice. TSS, transcriptional start site.

Supplementary Figure S2 **Identification and metabolic indexes of Mettl^3cO^E mice. A**. Schematics of vectors for conditional overexpression of *Mettl3* (pX602-AAV8-LP1-Mettl3, Mettl3^cOE^) and Mut-*Mettl3* (pX602-AAV8-LP1-Mut-*Mettl3*, Mettl3^cOE^-Mut). Mettl3^cOE^-Mut mice served as the control for the following experiments. **B**. Living imaging demonstrating luciferase specifically expressed in Mettl3^cOE^ and Mettl3^cOE^-Mut mouse livers. The color-coded shapes represent luciferase signals. **C**. qRT-PCR validation of *Mettl3* and Mut-Mettl3 conditional overexpression in Mettl3^cOE^ and Mettl3^cOE^-Mut mouse livers. *n* = 3. **D.** Western blotting detection and quantification of the expression of Mettl3 and mutant *Mettl3* proteins in liver extracts from wild-type (WT), Mettl3^cOE^, and Mettl3^cOE^-Mut mice. Actin served as the loading control, *n* = 3. **E**. Fat volume of Mettl3^cOE^-Mut and Mettl3^cOE^ mice after 7 weeks of HFD treatment. *n* = 10. Data are presented as mean ± SEM. Significant difference was determined by unpaired student’s t-test (**, *P* < 0.01; ***, *P* < 0.001; ****, *P* < 0.0001). ITR, inverted terminal repeats. Raw data are displayed in Table S2.

Supplementary Figure S3 Identification and metabolic indexes of Mettl3cKO mice. **A**. Diagram illustrating the procedure of generating *Alb*-Cre-mediated *Mettl3* conditional knockout mice. **B**. PCR confirmed the conditional knockout of Mettl3 exons 2–4 in the livers of *Mettl3*^flox/flox^; *Alb*-Cre (Mettl3^cKO^) mice. **C**. qRT-PCR showing the decrease of *Mettl3* mRNA in Mettl3^cKO^ mouse liver. *n* = 3. **D**. Western blotting detection and quantification of Mettl3 protein expression in the liver extracts from Mettl3^Ctrl^ and Mettl3^cKO^ mice. Tubulin served as the loading control. *n* = 3. **E**. Immunostaining of Mettl3 (Green) in Mettl3^Ctrl^ and Mettl3^cKO^ mouse livers. Scale bar, 50 μm. **F**. Fat volume of Mettl3^Ctrl^ and Mettl3^cKO^ mice after 20 weeks of HFD treatment. *n* = 10. Data are presented as mean ± SEM. Significant difference was determined by unpaired student’s *t*-test (***, *P* < 0.001; ****, *P* < 0.0001). Raw data are displayed in Table S2.

Supplementary Figure S4 **m^6^A pattern in liver of Mettl3^cKO^ mouse after HFD. A**. m^6^A consensus motif in mRNAs from liver of Mettl3^cKO^ mouse after 20 weeks HFD treatment. **B**. Transcriptome-wide distribution of m^6^A sites on mRNAs from liver of Mettl3^cKO^ mouse after 20 weeks HFD treatment. Pie chart showing the proportion of m^6^A sites in distinct non-overlapping segments: 5ʹ UTR, TSS, CDS, stop codon, and 3ʹ UTR. **C**. Percentage of m6A-methylated mRNAs with different numbers of m^6^A sites in Mettl3^cKO^(HFD) mouse liver.

Supplementary Table S1 **The identified m^6^A sites in Mettl3^Ctrl^(ND), Mettl3^Ctrl^(HFD), and** Mettl3^cKO^**(HFD) mice**.

Supplementary Table S2 **Statistics source data**.

Supplementary Table S3 **The information of primers****used in this study.**
