# Supplementary figures and images for: m6A Regulates Liver Metabolic Disorders and Hepatogenous Diabetes
Source: Genomics Proteomics Bioinformatics. 2020 Nov 5;18(4):371–83. doi: 10.1016/j.gpb.2020.06.003 (PMC8242261; doi:10.1016/j.gpb.2020.06.003)

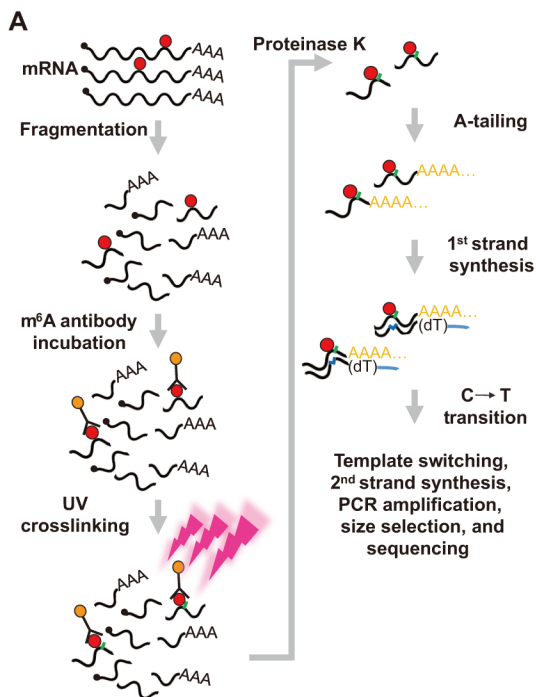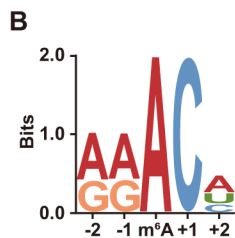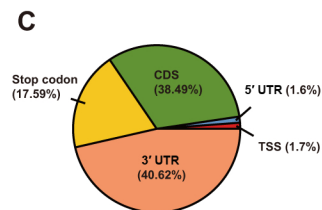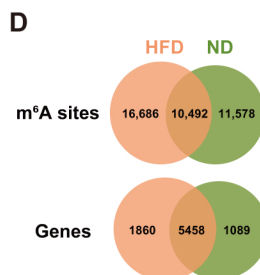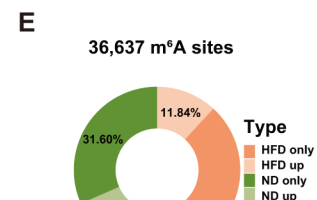

Supplement: Supplementary Figure S1 — m6A pattern in HFD mouse liver. A. Schematic illustration of miCLIP library construction procedure. B. m6A consensus motif in mRNAs from HFD mouse liver. C. Transcriptome-wide distribution of m6A sites. Pie chart shows the proportion of m6A sites in distinct non-overlapping segments: 5′ UTR, TSS, CDS, stop codon, and 3′ UTR. D. Venn diagram depicting the number of unique and overlapping m6A sites on liver mRNAs from ND and HFD mice, as well as the number of unique and overlapping methylated genes in ND and HFD mouse livers. Numbers represent the counts of m6A sites or methylated genes in each group. E. Donut chart showing the proportion of unique m6A sites (only) and overlapping m6A sites with higher level (up) in the livers of ND and HFD mice. TSS, transcriptional start site. [file mmc1.pdf]

**A**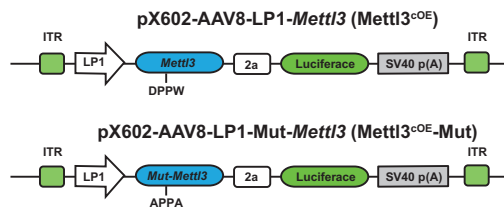**B**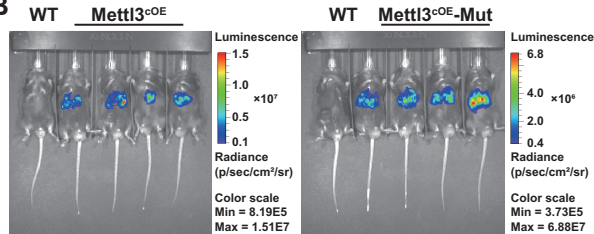**C**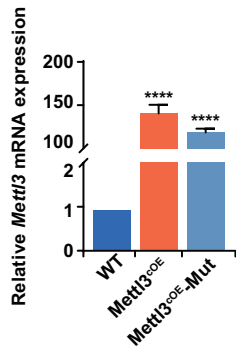**D**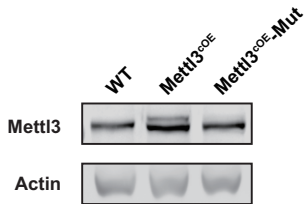**Relative *Mettl3* protein expression**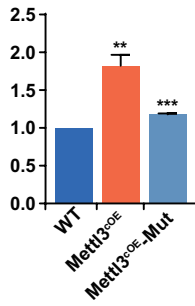**E**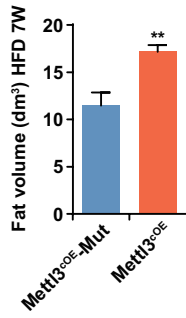

Supplement: Supplementary Figure S2 — Identification and metabolic indexes of Mettl3cOE mice. A. Schematics of vectors for conditional overexpression of Mettl3 (pX602-AAV8-LP1-Mettl3, Mettl3cOE) and Mut-Mettl3 (pX602-AAV8-LP1-Mut-Mettl3, Mettl3cOE-Mut). Mettl3cOE-Mut mice served as the control for the following experiments. B. Living imaging demonstrating luciferase specifically expressed in Mettl3cOE and Mettl3cOE-Mut mouse livers. The color-coded shapes represent luciferase signals. C. qRT-PCR validation of Mettl3 and Mut-Mettl3 conditional overexpression in Mettl3cOE and Mettl3cOE-Mut mouse livers. n = 3. D. Western blotting detection and quantification of the expression of Mettl3 and mutant Mettl3 proteins in liver extracts from wild-type (WT), Mettl3cOE, and Mettl3cOE-Mut mice. Actin served as the loading control, n = 3. E. Fat volume of Mettl3cOE-Mut and Mettl3cOE mice after 7 weeks of HFD treatment. n = 10. Data are presented as mean ± SEM. Significant difference was determined by unpaired student’s t-test (**, P < 0.01; ***, P < 0.001; ****, P < 0.0001). ITR, inverted terminal repeats. Raw data are displayed in Table S2. [file mmc2.pdf]

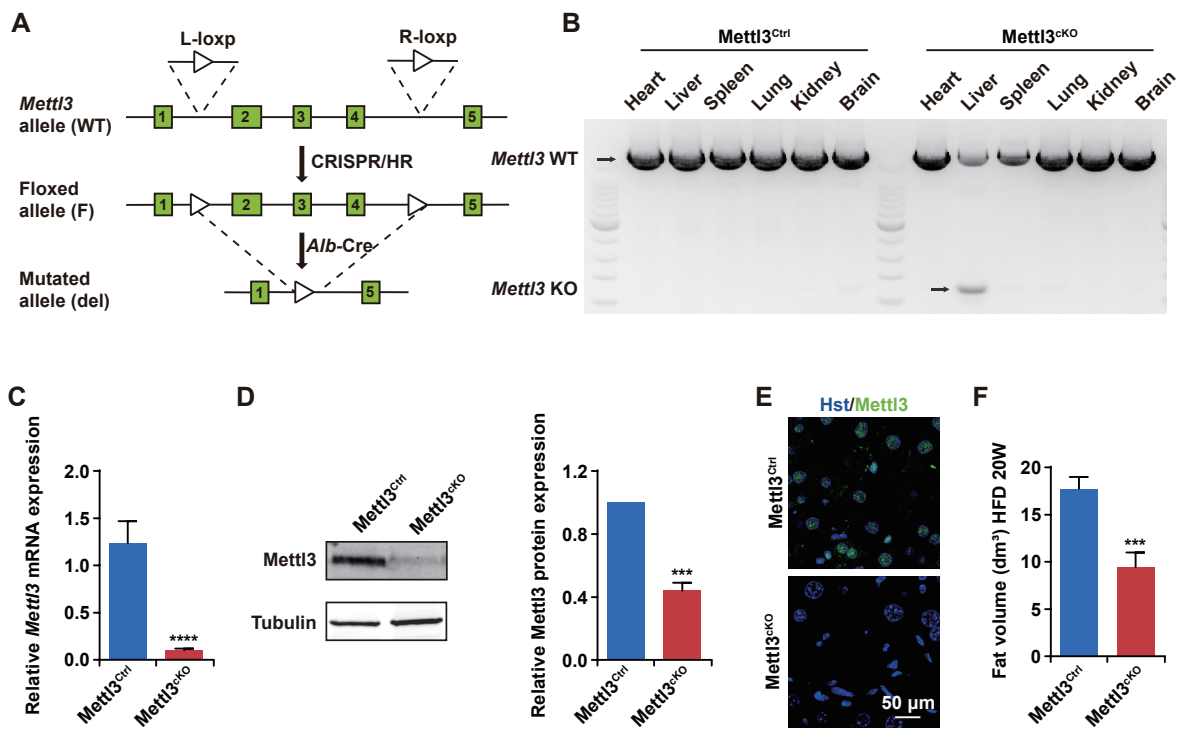

Supplement: Supplementary Figure S3 — Identification and metabolic indexes of Mettl3cKO mice. A. Diagram illustrating the procedure of generating Alb-Cre-mediated Mettl3 conditional knockout mice. B. PCR confirmed the conditional knockout of Mettl3 exons 2–4 in the livers of Mettl3flox/flox; Alb-Cre (Mettl3cKO) mice. C. qRT-PCR showing the decrease of Mettl3 mRNA in Mettl3cKO mouse liver. n = 3. D. Western blotting detection and quantification of Mettl3 protein expression in the liver extracts from Mettl3Ctrl and Mettl3cKO mice. Tubulin served as the loading control. n = 3. E. Immunostaining of Mettl3 (Green) in Mettl3Ctrl and Mettl3cKO mouse livers. Scale bar, 50 μm. F. Fat volume of Mettl3Ctrl and Mettl3cKO mice after 20 weeks of HFD treatment. n = 10. Data are presented as mean ± SEM. Significant difference was determined by unpaired student’s t-test (***, P < 0.001; ****, P < 0.0001). Raw data are displayed in Table S2. [file mmc3.pdf]

**A**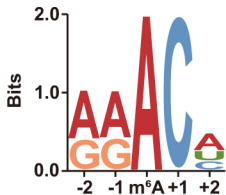**B**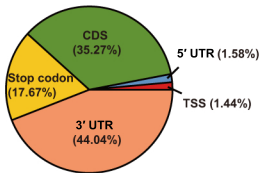**C**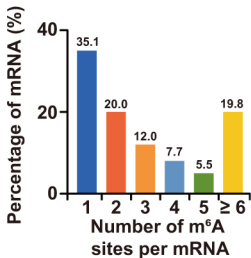

Supplement: Supplementary Figure S4 — m6A pattern in liver of Mettl3cKO mouse after HFD. A. m6A consensus motif in mRNAs from liver of Mettl3cKO mouse after 20 weeks HFD treatment. B. Transcriptome-wide distribution of m6A sites on mRNAs from liver of Mettl3cKO mouse after 20 weeks HFD treatment. Pie chart showing the proportion of m6A sites in distinct non-overlapping segments: 5ʹ UTR, TSS, CDS, stop codon, and 3ʹ UTR. C. Percentage of m6A-methylated mRNAs with different numbers of m6A sites in Mettl3cKO(HFD) mouse liver. [file mmc4.pdf]
